# COVID-19, Endothelium and the Cardiometabolic Patient: A Possible Role for Capillary Leak Syndrome

**DOI:** 10.3390/biomedicines10102379

**Published:** 2022-09-23

**Authors:** Vaia Lambadiari, Emmanouil Korakas, Evangelos Oikonomou, Evanthia Bletsa, Aikaterini Kountouri, Athina Goliopoulou, Ignatios Ikonomidis, Gerasimos Siasos

**Affiliations:** 1 2nd Department of Internal Medicine, Attikon University Hospital, National and Kapodistrian University of Athens, Medical School, 12462 Athens, Greece; 2 3rd Department of Cardiology, National and Kapodistrian University of Athens, Medical School, Sotiria Chest Disease Hospital, 11527 Athens, Greece; 3 Cardiometabolic Disease Unit, 3rd Department of Cardiology, National and Kapodistrian University of Athens, Medical School, Sotiria Chest Disease Hospital, 11527 Athens, Greece; 4 Laboratory of Preventive Cardiology, Second Cardiology Department, Attikon University Hospital, National and Kapodistrian University of Athens, Medical School, 12462 Athens, Greece

**Keywords:** COVID-19, capillary leak syndrome, albumin, endothelium, cardiometabolic, inflammation

## Abstract

Capillary leak syndrome is an under-diagnosed condition leading to serious hypoalbuminemia with diffuse edema, pulmonary edema, severe hypotension, and possibly death. Sepsis leading to hemophagocytic lymphohistiocytosis (HLH) is a major risk factor; however, capillary hyper-permeability is the core underlying pathophysiological mechanism. Endothelial dysfunction plays a major role in cardiometabolic disease through insulin resistance, lipotoxicity, and, eventually, oxidative stress and chronic inflammation. We review the literature concerning the aforementioned mechanisms as well-established risk factors for adverse COVID-19 outcomes. We especially focus on data regarding the underlying endothelial effects of SARS-CoV-2 infection, including direct damage and increased vascular leakage through a hyper-inflammatory cascade and diminished nitric oxide bioavailability. Interestingly, an increased incidence of hypoalbuminemia has been observed in patients with severe COVID-19, especially those with underlying cardiometabolic disease. Importantly, low albumin levels present a strong, positive association with poor disease outcomes. Therefore, in this review article, we highlight the important role of cardiovascular risk factors on endothelium integrity and the possible link of endothelial damage in the hypoalbuminemia-associated adverse prognosis of COVID-19 patients.

## 1. Introduction

Coronavirus disease 19 (COVID-19), caused by the betacoronavirus SARS-CoV-2, is the greatest challenge for global health systems in the last century, with its death toll already surpassing 5 million deaths. The clinical presentation varies from mild or even asymptomatic disease to severe respiratory infection, with acute respiratory distress syndrome (ARDS) and respiratory failure [1]. However, since the report by Varga et al. [2], where postmortem histology of three patients with severe infection revealed inflammation of the endothelial cells in different vascular beds, a growing body of evidence has pointed towards endothelial dysfunction as a major driver of cardiovascular events and, consequently, higher morbidity and mortality in patients with COVID-19 [1]. Diabetes mellitus, obesity, and hypertension, as major components of cardiometabolic syndrome, are also associated with endothelial dysfunction, and they have emerged as primary risk factors for aggravated COVID-19 outcomes [3,4]. In a meta-analysis, the mortality of hospitalized diabetic patients compared to non-diabetic ones was 21.3% vs. 6.1% (OR:2.39), and similar results were shown regarding the severity of the disease (OR: 1.43) [5]. Similarly, the risk of hospitalization, requiring intensive care, and in-hospital mortality was higher in obese COVID-19 patients compared to non-obese patients [6]. The molecular and pathophysiological mechanisms of these associations are various. Chronic, low-grade inflammation, increased oxidative stress, immune defects, and endothelial derangement with coagulopathy and hyperpermeability have been proposed to exacerbate the fulminant hypercytokinemia that accompanies COVID-19 disease and lead to sepsis-like manifestations and multi-organ failure [1].

Capillary leak syndrome refers to a constellation of clinical features, including serious hypoalbuminemia with diffuse edema, exudative serous cavity infusions, and hypotension [7]. Its true incidence is unknown, possibly due to under-diagnosis; however, it can complicate the natural course of various conditions such as sepsis, autoimmune diseases, hemophagocytic lymphohistiocytosis (HLH), and viral hemorrhagic fevers, or it can be idiopathic (Clarkson’s disease). Regardless of the etiology, the underlying pathophysiological mechanism involves increased capillary permeability to proteins and their subsequent extravasation to the interstitial space. Significant hypoalbuminemia has been a frequent finding in severe COVID-19 patients, with a recent meta-analysis revealing that hypoalbuminemia was associated with poor prognosis in terms of severity and mortality [8]. Even more importantly, this association has been found to be independent of hepatic function, implying a pivotal role for the deranged endothelial barrier as the trigger. Therefore, it has been implied that capillary leak may be one of the main pathophysiological mechanisms for worse outcomes in cardiometabolic patients, where underlying endothelial dysfunction already exists. In this review, the main effects of cardiometabolic disease and COVID-19 on endothelium will be discussed, focusing especially on the continuum from metabolic disease to endothelial impairment, capillary leak syndrome, and the prognosis of patients with COVID-19.

## 2. Endothelial Dysfunction in the Cardiometabolic Patient

**Figure 1:**
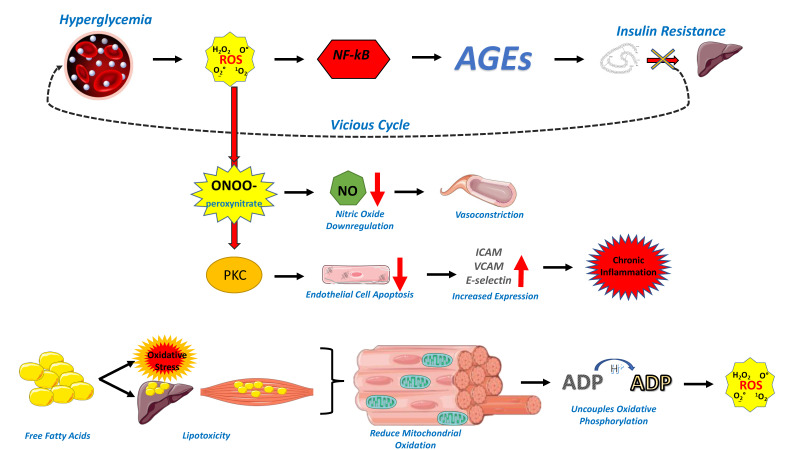
Cellular and biochemical mechanisms causing endothelial damage in patients with cardiometabolic disease. ROS; reactive oxygen species, NF-kB; nuclear factor kappa B, AGEs; advanced glycation end products, NO; nitric oxide, PKC; protein kinase c, ICAM; intracellular adhesion molecule, VCAM; vascular cell adhesion molecule, ADP; Adenosine diphosphate.

Cardiometabolic disease is a constellation of clinical entities, which include insulin resistance and overt diabetes mellitus, obesity, dyslipidemia, hypertension, and coronary artery disease. The common ground between these disorders is endothelial dysfunction, which may be attributed to genetic and environmental factors, but it also shares various mechanisms with metabolic derangement, especially insulin resistance [9], as presented in Figure 1. Hyperglycemia increases the production of reactive oxygen species (ROS), inducing a state of increased oxidative stress, which, in turn, leads to activation of the pro-inflammatory NF-kB pathway and increased production of advanced glycation end products (AGEs), which lead to aggravated insulin resistance in a vicious cycle [10]. Interestingly, insulin infusion can substantially increase left ventricular ejection fraction (LVEF) in response to submaximal dynamic exercise among both healthy subjects and patients with diabetes [11]. The rise in exercise-LVEF on insulin is likely attributed to an enhancement of ventricular contractility. Nevertheless, patients with diabetes have impaired diastolic function both at rest and during exercise when compared to healthy controls, while the insulin-induced rise in LVEF is significantly lower in those patients when compared to healthy subjects due to insulin resistance [11]. Similarly, obese and overweight patients have impaired diastolic function, and insulin-induced increase in LVEF is lower in those patients when compared to subjects with normal weight [12]. These data imply metabolic pathogenesis for the impaired left ventricular function in diabetes and obesity.

To our knowledge, insulin resistance and endothelial dysfunction share many crosstalk metabolic pathways. As a result, insulin resistance often co-exists with endothelial dysfunction in CVD. Insulin resistance induces inflammation, vasoconstriction, and vascular smooth cell proliferation via the mitogen-activated protein kinase/extracellular signal-regulated kinase (MAPK/ERK) pathway [9]. Indeed, a decreased sensitivity to the normal vascular actions of insulin impedes nitric oxide (NO) production and bioability due to oxidative burden, thus playing an additional important role in the development of endothelial dysfunction in states of insulin resistance [13]. Furthermore, insulin resistance provokes vascular dysfunction via the abnormal production and activation of the vasoconstrictor/proatherogenic peptide endothelin-1, pro-inflammatory adipokines, and elevated levels of free fatty acids by adipose tissue [14].

Oxidative stress leads to the production of peroxynitrite, which downregulates the availability of NO and therefore promotes vasoconstriction [15], while ROS and the activated protein kinase C (PKC) accelerate the apoptosis of endothelial cells and enhance the expression of intercellular adhesion molecule (ICAM), vascular cell adhesion molecule (VCAM), and E-selectin, leading to chronic inflammation and hypercoagulability [16]. On the other hand, AGEs disrupt the structure of extracellular matrix proteins, such as collagen, and promote the transformation of infiltrated macrophages to foam cells, increasing pro-inflammatory molecules, which, in turn, promote inflammation and hyperpermeability of the vessel wall [17].

Apart from glucotoxicity, the increase in free fatty acids (FFAs) in insulin-resistant states such as diabetes and obesity leads to oxidative stress and lipotoxicity through several pathways [9]. The increased intramyocellular lipids reduce mitochondrial oxidation, and this mitochondrial derangement uncouples oxidative phosphorylation and increases the production of ROS while downregulating potent antioxidants such as superoxide dismutase (SOD). The pro-inflammatory NF-kB pathway is activated, and this leads to increased expression of pro-inflammatory cytokines, such as interleukin 6 (IL-6) and tumor necrosis factor-α (TNF-α) [9,18]. Ceramide, a by-product of long-chain saturated fatty acids (SFAs), reduces the availability of NO and increases ROS production [19]. Once again, ROS upregulates peroxynitrite production and prevents the dilatation of the arterial wall, while insulin signaling in the endothelial cells is impaired, further aggravating insulin resistance and inflammation. Chronic inflammation inhibits the expression of endothelial nitric oxide synthase (eNOS) while it enhances the expression of adhesion molecules and endothelin 1 (ET-1), further disrupting endothelial integrity [20].

Endothelial dysfunction seems to be at the core of the pathophysiological processes underlying hypertension [21]. Increased oxidative stress through the overproduction of ROS leads to vasoconstriction and vascular hypertrophy, while increased lipid peroxidation accelerates vascular aging. Higher levels of pro-inflammatory cytokines, such as C-reactive protein (CRP), downregulate eNOS activity. An impaired immune response is also involved in the process of endothelial repairing; in hypertensive patients, increased angiotensin-2 or aldosterone directly activates the innate immune system, which can result in inappropriate autoimmune responses against vascular wall antigens and ROS production [22]. Furthermore, hypertension is often accompanied by insulin resistance and obesity, sharing the above-stated mechanisms of endothelial dysfunction. In fact, dysregulation of adipokines in obesity, such as hyperleptinemia and hypoadiponectinemia, downregulate NO production and enhance MCP-1 expression and seem to be a major factor for the generation of hypertension in obese patients while further enhancing leukocyte infiltration [23]. Perivascular adipose tissue (PVAT), despite normally exerting anticontractile effects, promotes vasoconstriction and endothelial dysregulation in obesity by upregulating the secretion of pro-inflammatory cytokines while downregulating NO production [24]. Finally, in the setting of activated endothelium with overexpression of adhesion molecules, platelets are also activated and promote thrombosis, while increased values of vascular endothelial growth factor (VEGF) and ET-1 lead to abnormal angiogenesis and endothelial dysfunction as well [25]. Notably, both classic and novel anti-diabetic agents seem to enhance endothelial function and arterial wall properties via several molecular pathways, such as inhibition of inflammation and oxidative burden [26,27]. They offer substantial cardioprotective properties beyond the field of hyperglycemia and insulin resistance, thus playing a major role in the prevention of cardiovascular disease. Indeed, gliflozines exert direct anti-atherogenic effects in cultured human umbilical vein endothelial cells (HUVECs) through modification of TNF-α release and the consequent downregulation of adhesion molecules. Beyond endothelial function, gliflozines also improve the integrity of the endothelial layer, as it can be estimated based on endothelial glycocalyx thickness [28]. Additionally, metformin may activate AMPK [29] and subsequently eNOS and NO release promoting the health of the vessel wall [30]. Furthermore, metformin attenuates the production of oxidized lipids through the beneficial modification of the oxidative milieu [31]. Regarding the effect of glucagon-like peptide 1 receptor agonists (GLP-1RAs) and dipeptidyl peptidase 4- inhibitors (DDP-4is) on endothelial function, studies show heterogeneity [32]. Improvement in FMD with GLP-1RAs may be attributed to the regulation of hyperglycemia, especially when patients are naïve to other anti-diabetic medications and to the modification of the circulation markers of oxidative status [33]. 

## 3. Endothelial Dysfunction in COVID-19

Early in the pandemic, a high incidence of cardiovascular events was noted in COVID-19 patients, especially those with severe disease [1]. Among risk factors for adverse clinical outcomes such as age, male gender, smoking history, and malignancies, metabolic diseases showed a remarkable and independent association, with ORs ranging from 1.9 to 2.68 for diabetes and as high as 5.70 for obesity [34]. Chronic inflammation, with higher levels of pro-inflammatory mediators, and immune dysregulation, including suppressed chemokine responses, inappropriate T_reg_ and T_h_17 activation, and neutrophil extracellular traps (NETs) dysfunction, have a well-established association with the exacerbation of COVID-19 hyper-inflammatory response; however, the underlying endothelial dysfunction seems to be the key player.

SARS-CoV-2 affects the endothelium in several ways [1]. Direct action on the endothelium was supported by the first report by Varga et al. [2], where viral particles were found in glomerular cells, and the results were similar in other reports where different vascular beds were involved [35,36]. In a case series including 64 patients, viral RNA was localized by in situ hybridization in endothelial cells in the lung, kidney, heart, brain, and other organs [37]. SARS-CoV-2 enters host cells through binding to the ACE2 receptor; however, this leads to a downregulation of ACE2 expression [38]. In a recent meta-analysis that included 1471 COVID-19 patients, lower subcutaneous adipose tissue ACE2 expression, measured by RNA sequencing, had an inverse association with the risk of severe disease in cardiometabolic patients [39]. Under normal circumstances, ACE2 counteracts the effects of the renin–angiotensin–aldosterone system (RAAS) by driving the degradation of angiotensin I and angiotensin II to angiotensin 1–9 and finally to angiotensin 1–7, which have vasodilatory and anti-inflammatory effects [40]. In COVID-19 disease, the decreased levels of angiotensin 1–7 and increased levels of angiotensin II lead to lower NO bioavailability due to a downregulation of eNOS activity. Angiotensin II further aggravates endothelial dysfunction by promoting ROS and IL-6 production [41,42], while it also enhances plasminogen-activator inhibitor type 1 (PAI-1) production and, therefore, inhibits fibrinolysis and the consequent dissolution of thrombi [43]. Overall, the damage of endothelial cells leads to increased tissue factor expression, upregulation of adhesion molecules, and neutrophil recruitment with the release of NETs, inducing further damage and activating the coagulation cascade [44].

Vascular leakage is a pivotal mechanism that leads to pulmonary congestion or even pulmonary edema and generalized hypoalbuminemia, which are observed in many severe COVID-19 patients. Direct infection of endothelial cells leads to serious endothelitis, which eventually results in cell lysis and death, damaging the integrity of the vessel wall [2]. The decreased ACE2 levels and the subsequent increase in angiotensin II levels indirectly affects the bradykinin–kallikrein pathway by enhancing the bradykinin receptor B1 activity, and this action leads to increased endothelial permeability [45]. Immune cells, such as the recruited neutrophils, pro-inflammatory cytokines, such as IL-1β and IL-6, and vasoactive molecules, such as bradykinin and thrombin, lose inter-endothelial junctions and enhance cell contractility, leading to gaps in the endothelial barrier and increased extravasation [46]. Finally, inflammatory mediators activate glucoronidases and lead to the degradation of the endothelial glycocalyx [47]. Indeed, elevated serum levels of components of the endothelial glycocalyx, such as syndecan and hyaluronic acid, have been found in patients with severe COVID-19, indicating increased damage to its structural integrity. In a recent study by Lambadiari et al. [48], including 70 patients 4 months after COVID-19 infection, it was shown that COVID-19 patients had higher perfused boundary region (PBR) of the sublingual arterial microvessels compared to controls (2.07 ± 0.15 µm vs. 1.89 ± 0.17 µm, *p* = 0.001) and similar to hypertensive patients (2.07 ± 0.15 µm vs. 2.07 ± 0.26 µm, *p* = 0.8), along with higher levels of malondialdehyde (MDA) and thrombomodulin, implying chronic endothelial hyperpermeability due to oxidative stress. These deleterious results in endothelial function are not temporary; in the same patient group, PBR values were worse at 12-month follow-up [49]. As the role of the endothelial glycocalyx is well-established in cardiometabolic disease and its complications, its value as a risk factor for COVID-19 hypoalbuminemia and thrombo-inflammation in these patients is undeniable and will be further consolidated by future studies.

## 4. Capillary Leak Syndrome

Capillary leak syndrome is defined as the combination of severe hypoalbuminemia with diffuse pitting edema, exudative serous cavity effusions, non-cardiogenic pulmonary edema, and hypotension which, in the most serious cases, can progress to resistant hypovolemia and shock [7,50,51]. Its incidence depends on the underlying cause; however, as these symptoms are frequently encountered in the course of many diseases, the syndrome is often underdiagnosed. Regardless of the etiology, however, the exaggerated endothelial permeability to proteins lies in the center of its pathophysiology [52], which, in turn, seems to be the result of an increase in pro-inflammatory cytokines in most cases. Cadherin is a fundamental component of adherent junctions, the main type of junctions through which endothelial cells bind to neighboring cells. Mild inflammatory stimuli cause cadherin internalization, weakening these junctions; as inflammation proceeds, adherent junctions are further disrupted, leading to gaps between endothelial cells and a massive increase in protein permeability [53]. In a study by Atkinson et al., it was shown that, in the acute phase of the syndrome, substances of up to 900 kDa in molecular weight were extravasated into the interstitial space; under physiological conditions, the endothelial barrier is permeable to less than 5% of albumin, with a molecular weight of only 66.5 kDa [54].

Many diseases have been associated with capillary leak syndrome, including certain drugs, hemorrhagic fevers, ovarian hyperstimulation syndrome, and others [7]. However, sepsis leading to hemophagocytic lymphohistiocytosis (HLH) is the most prominent risk factor, and it seems that this is the case in severe COVID-19 disease. HLH is a condition that affects multiple systems and is characterized by an aberrant, uncontrollable immune response, which results in increased levels of pro-inflammatory cytokines such as IL-6, IL-2, (MCP-1), and TNF-α [55,56,57]. In COVID-19 infection, the virus binds to the ACE2 receptor in type I and type II alveolar epithelial cells in the lungs and then enters the cells through serine proteases such as transmembrane Serine Protease 2 (TMPRSS2). This enhances cell apoptosis and the production of inflammatory mediators; at the same time, increased apoptosis of lymphocytes leads to lymphocytopenia [58]. In addition, the apoptosis of alveolar epithelial cells leads to activation of the NOD-like receptor family pyrin domain-containing 3 (NLRP3) inflammasome [59]. The combined effect of these actions is a fulminant hypercytokinemia known as “cytokine storm”, a state which resembles HLH. In people with diabetes, the already pre-existing increased pro-inflammatory cytokine profile and oxidative stress could further aggravate the already aberrant immune response to COVID-19, while the immunosuppression per se could facilitate viral entry into endothelial cells [34]. Innate and adaptive immunity are compromised in diabetes and obesity, with an imbalance of CD4+ T helper cells toward Th17 and Th22 pro-inflammatory subsets and a persistent NET formation after infection, which prolongs and enhances the inflammatory cascade [60]. Finally, NLRP3 expression is increased in insulin-resistant states through the activation of the NF-kB pathway, which is combined with the release of C3a and C5a anaphylatoxins during SARS-CoV-2 infection [59].

Idiopathic systemic capillary leak syndrome (ISCLS—Clarkson’s disease) is another type of syndrome which seems to have an association with some cases of COVID-19 infection. First described in 1960 [61], it is an extremely rare disease, with about 260 cases reported worldwide until recently [62]. It is characterized by recurrent flares, with the trigger behind them not always being clear; however, inflammation has a robust association, and viral infections, such as influenza and respiratory syncytial virus, have been recognized in about half of the cases in recent cohorts [63]. With general, flu-like preceding symptoms being a frequent but inconsistent finding, patients with ISCLS rapidly develop the SCLS diagnostic triad, composed of the “3 H’s”: hypotension, hemoconcentration (hematocrit > 49–50% in men, 43–45% in women), and hypoalbuminemia (<3.0 g/dL), with shock and generalized edema due to huge plasma extravasation [62]. After a mean period of 1–3 days, the capillary endothelial barrier is spontaneously restored, with rapid fluid remobilization into the intravascular space, an effect that is often fatal due to subsequent pulmonary edema. Other causes of death include multi-organ failure due to hypovolemic shock and thromboembolic events due to hyperviscosity and hypercoagulability. While about 80% of the patients have a history of monoclonal gammopathy of undetermined significance (MGUS) [64], the pathogenetic role of the monoclonal protein is not clearly defined. In fact, vascular endothelial hyper-permeability is the main pathogenetic mechanism of the disease, although persistent structural abnormalities have not been found in biopsy specimens, indicating a reversible defect in endothelial integrity [62]. Elevated levels of pro-inflammatory cytokines such as CXCL-10 and IL-6 have been found in the acute serum of patients with ISCLS, and incubation of microvascular endothelial cells with acute serum reduced cadherin expression and thus led to a loosening of adherent junctions and endothelial gaps [65,66]. This effect implied the presence of a soluble factor that is responsible for the endothelial disruption, with possible candidates being VEGF and angiopoietin-2 (Ang-2), whose levels are elevated during disease flares [65,67]. These factors contribute to endothelial permeability in states such as sepsis [68,69,70]; at the same time, their levels are increased in diabetes and obesity and contribute to complications such as nephropathy and retinopathy, where endothelial hyperpermeability and degradation of the endothelial glycocalyx is a key factor [71,72]. Although an association between metabolic diseases and ISCLS has not been recognized, it is possible that the chronic endothelial dysfunction in these states could be a favorable background in a hyperinflammatory state like COVID-19.

## 5. Capillary Leak Syndrome in the Cardiometabolic COVID-19 Patient

Many studies have shown an increased incidence of hypoalbuminemia in severe COVID-19 patients; in fact, low albumin levels have a strong, positive association with poor disease outcomes [73]. Since the first months of the pandemic, a strong correlation between low albumin levels and adverse clinical outcomes was shown [73], and in a more recent meta-analysis by Soetedjo et al. [8], hypoalbuminemia was a risk factor for poor prognosis (OR: 6.97). While the mechanisms linking hypoalbuminemia with an adverse prognosis of patients with COVID-19 have not been directly addressed, several mechanisms have been proposed. In patients with sepsis, albumin modulates inflammatory status and oxidative damage and may also regulate effective plasma volume, circulation, and systematic perfusion [74,75,76]. Low albumin levels are also associated with lower oxygenation, especially in patients with acute respiratory distress syndrome [77].

However, the mechanisms underlying hypoalbuminemia in patients with COVID-19 are not fully elucidated. It has been demonstrated that impaired liver synthesis was not the trigger, as hypoalbuminemia occurred within the first 3 days of patients’ admission, far shorter than the half-life of serum albumin, while albumin concentrations were inversely correlated with inflammation markers such as CRP, suggesting that systemic inflammation and capillary leak syndrome was the main pathogenetic mechanism [78]. Taking into account the previously described correlations between metabolic disease, chronic endothelial dysfunction, and COVID-19, it would be expected that hypoalbuminemia could have a positive association with the presence of cardiometabolic co-morbidities in these patients. Indeed, in the study by Wu et al., median serum albumin concentrations were significantly low in patients requiring intermediate or intensive care hospitalization (20 g/L and 28 g/L, respectively), with the median BMI in both groups being 27.8 kg/m^2^ and 26.6 kg/m^2^, respectively; even more importantly, the presence of diabetes was associated with a higher rate of ICU admission [79]. No other significant correlations were found for other co-morbidities, implying that the endothelial dysfunction of insulin-resistant states was crucial in hyperpermeability and disease severity. In another cohort, hypertension was found in 38.7% of the patients with hypoalbuminemia compared to 17.1% in patients with normal albumin levels (*p* < 0.001), and the results were similar for the presence of diabetes (22.6% vs. 5.7%, *p* < 0.001) [78]. As in the previous study, no association was established with impaired liver function tests, pointing towards capillary leak as the main trigger. Serum albumin levels < 30 g/L were found in almost half of COVID-19 hospitalized patients in a report by Bassoli et al. [80]. Hypertension was present in 43.8% in the group with albumin < 30 g/L, compared to 24.5% in those patients with higher levels (*p*: 0.003); on the contrary, no such association was noted for diabetes, although it must be noted that its incidence was considerably lower in the total cohort. A statistically significant association between hypoalbuminemia and BMI, but not for diabetes, was found by Hundt et al., again independent of abnormal liver function [81]. Chen et al. in 482 hospitalized patients where hypoalbuminemia had a ratio of 53.7%, showed a statistically significant higher prevalence of diabetes in the hypoalbuminemia group, while hypertension was also more frequent, albeit marginally insignificant [82]. Even more interestingly, Viana et al. showed that hypoalbuminemia on admission (<34 g/L) was more frequent in COVID-19 non-survivors than survivors, and the same applied to the incidence of diabetes, hypertension, and dyslipidemia, all hallmarks of cardiometabolic disease [83]. In addition, serum albumin concentration showed linear correlation with highest hs-CRP (r = −0.306, *p* < 0.001), highest procalcitonin (r = −0.286, *p* < 0.001), and lowest lymphocyte count (r = 0.264, *p* < 0.001), indicating a clear link with the hyperinflammatory response in COVID-19, especially in the setting of chronic inflammation in metabolic disease. The fact that hypoalbuminemia was associated with mortality irrespectively of hs-CRP levels further indicates that albumin is not just a biomarker used in risk stratification but that capillary leak and the subsequent hypoalbuminemia and thrombo-inflammation have a direct pathogenic role in severe COVID-19 cases. Finally, in a report by Hirashima et al., albumin was significantly lower in critical and severe COVID-19 patients compared to milder cases, and hypertension and diabetes were again more frequent in the first group (*p*: 0.014 and <0.001, respectively) in the absence of any liver disorders [84]. Contrary to these results, the strong correlation of capillary leak syndrome in COVID-19 patients to the presence of cardiometabolic disorders has not been indicated in some other reports, where hypoalbuminemia was consistently associated with increased disease severity [8,85,86]; however, this possibly only implies that COVID-19 infection exerts its deleterious effects with several other immune and inflammatory mechanisms, with capillary leak being crucial, but not the only one. These findings are summarized in Table 1 and Table 2.

Apart from incident hypoalbuminemia in the course of severe disease, another interesting link between cardiometabolic co-morbidities and capillary leak in COVID-19 is indicated through several SCLS cases. Pineton de Chambrun et al. were the first to report a fatal relapse of SCLS during a SARS-CoV-2 infection in a patient with a history of MGUS [87], and Lacout et al. reported the first SCLS episode elicited by COVID-19 in a patient with no medical history [88]. Since then, a few other case reports have been published, mainly regarding patients with a known history of Clarkson’s disease [89,90,91,92,93,94,95]. However, in the report by Case et al., the patient’s medical history was important only for the presence of systemic hypertension [92]. Similarly, in the report by Cheung et al., a 59-year-old woman died due to acute SCLS, beginning 6 days after the onset of respiratory symptoms due to COVID-19 infection; her medical history again included only hypertension, with no known history of SCLS or previous episodes of peripheral edema [90]. The presence of hypertension was also noted in a 58-year-old man who also had an SCLS history [89], while, in the report by Beber et al., obesity and Sjogren’s disease were noted in the absence of any previous syndrome flares [93]. On the other hand, the incidence of SCLS episodes after COVID-19 vaccination is higher, especially with adenoviral vector vaccines [94]; however, although in the few case reports thoroughly described in the literature, a previous history of SCLS or MGUS is a frequent finding, medical history, and other details are not known for many other cases reported by national regulatory authorities. However, a case by Yatzusuka et al. must be noted where the patient had a medical history of hypertension, obesity, and psoriasis, with the first two conditions having a well-established role also in psoriatic exacerbations apart from their possible role in aggravating the COVID-19 endothelial dysfunction and the subsequent disease flare [95]. In general, the possible contribution of cardiometabolic disease to the capillary leak in COVID-19-mediated SCLS flares is not yet robust, albeit notable. A possible explanation for this might be the fact that COVID-19 flares and typical SCLS episodes have some remarkable differences; for example, SCLS is not generally characterized by detectable damage to endothelial cells, while, on the other hand, endothelial injury in COVID-19 might not always be the aftermath of capillary leak, but rather the result of thrombo-inflammation and hypercoagulability [79,85].

## 6. Conclusions

COVID-19 is a disease with multi-faceted clinical features, which vary from mild disease to fatal outcomes. The severity of the disease is associated with chronic low-grade inflammation and endothelial dysfunction underlying cardiometabolic diseases. Capillary leak, through endothelial hyperpermeability to proteins and the induction of pro-inflammatory, pro-coagulant pathways, has emerged as a pivotal pathogenetic factor of SARS-CoV-2 infection in cardiometabolic patients and as the major etiology of hypoalbuminemia in severe COVID-19 cases. A great body of evidence supports the association of this condition with underlying metabolic derangement, and few remarkable indications regarding the role of metabolic disease in the incidence of systemic capillary leak syndrome flares have been noted. Further studies are needed to consolidate these findings and enlighten the pathophysiology behind them; however, capillary leak seems to be the core process that needs to be addressed effectively in cardiometabolic patients with COVID-19 infection.

## Figures and Tables

**Table 1:** COVID-19 Prognosis and Serum Albumin Levels.

Author	Type of Study	Patients Characteristics	Main Findings
Paliogiannis et al. [73]	Systematic review and meta-analysis	⋅ 19,760 patients with COVID-19 ⋅ 6141 patients with high severity or poor outcomes	⋅ Hypoalbuminemia in patients with severe disease or adverse outcomes (SMD: −0.99 g/L, 95% CI, −1.11 to −0.88, *p* < 0.001).
Soetedjo et al. [8]	Systematic review and meta-analysis	⋅ 19 studies ⋅ 6200 patients with COVID-19	⋅ Hypoalbuminemia was associated with composite poor outcome [(OR 6.97, 95% CI, 4.20–11.55), I^2^ = 91.3%, *p* < 0.001 for all)].
Huang et al. [78]	Retrospective cohort study	⋅ 299 patients with COVID-19 ⋅ Age: 53.4 ± 16.7 years ⋅ Co-morbidities: 33.1%	⋅ Hypoalbuminemia in 35.5% of the patients. ⋅ 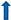 higher rates of hypoalbuminemia in non-survivors.
Wu et al. [79]	Retrospective cohort study	⋅ 174 patients with COVID-19 ⋅ 92 patients were admitted to IMW vs. 82 to the ICU ⋅ Male: 79.3% vs. 60.9% ⋅ Age: 62.5 (48.7–68) vs. 60 (49–73) years	⋅ Hypoalbuminemia in ICU compared to IMW patients [20 (18–23) vs. 28 (24–33) g L^−1^, *p* < 0.001]. ⋅ Hypoalbuminemia was associated with lower PaO_2_-to-FiO_2_ ratio, worse chest X-ray findings, and worse 30-day survival.
Bassoli et al. [80]	Retrospective cohort study	⋅ 207 patients with COVID-19 ⋅ Male: 68.6% ⋅ Age (IQR): 60.90 (49.17–71.29) years	⋅ Hypoalbuminemia in 50.7% of the patients. ⋅ Hypoalbuminemia was correlated with severity of COVID-19, death, CRP, and D-dimer.
Chen et al. [82]	Retrospective cohort study	⋅ 482 patients with COVID-19 ⋅ Male: 50.4% ⋅ Age (IQR): 56 (39–67) years	⋅ Hypoalbuminemia in 53.7% of the patients. ⋅ Hypoalbuminemia was associated with disease severity (OR 2.121, 95% CI, 1.258–3.577, *p* = 0.005).
Viana et al. [83]	Retrospective cohort study	⋅ 609 patients with COVID-19 ⋅ Male: 60.3% ⋅ Age (IQR): 71 (58–82) years	⋅ Hypoalbuminemia in 65.6% in non-survivors (vs. 38% in survivors). ⋅ Hypoalbuminemia was a predictor of mortality (HR 1.537, 95% CI 1.050–2.250, *p* = 0.027).
Hirashima et al. [84]	Retrospective cohort study	⋅ 61 patients with COVID-19 ⋅ Male: 65.6% ⋅ Age: 47.51 (20–88) years	⋅ 16 patients with critical or severe illness vs. 45 with moderate or mild symptoms. ⋅ Hypoalbuminemia among critically or severely ill patients.
Abdeen et al. [85]	Retrospective cohort study	⋅ 300 patients with COVID-19 ⋅ Male: 59.0% ⋅ Age: 61.5 ± 15.3 years	⋅ Hypoalbuminemia in patients with in-hospital mortality. ⋅ 73% reduction in mortality for each one-unit increase in albumin
Arnau-Barrés et al. [86]	Retrospective cohort study	⋅ 405 patients with COVID-19 ⋅ Male: 46.0% ⋅ Age: 79 ± 8.6 years	⋅ Hypoalbuminemia in non-survivors at baseline. ⋅ Severe hypoalbuminemia predicted in-hospital mortality (OR 2.18, 95% CI, 1.03–4.62, *p* = 0.039).

SMD; standard mean deviation, WBC; white blood cell, OR; odds ratio, ICU; intensive care unit, IMW; intermediate care ward, CRP; C-reactive protein.

**Table 2:** Hypoalbuminemia in COVID-19 and Cardiometabolic Risk Factors.

Author	Type of Study	Patient Characteristics	Cardiometabolic Risk Factors	Main Findings
Wu et al. [79]	Retrospective cohort study	⋅ 174 patients with COVID-19 ⋅ 92 patients were admitted to IMW vs. 82 to the ICU ⋅ Μale: 79.3% vs. 60.9% ⋅ Age: 62.5 (48.7–68) vs. 60 (49–73) years	⋅ ICU vs. IMW ⋅ BMI: 27.8 (25.4–31.9) vs. 26.6 (23.9–29.8) kg/m^2^ ⋅ Diabetes: 13.4 vs. 28.3%	⋅ Hypoalbuminemia was detected in both groups (20 g/L and 28 g/L, respectively). ⋅ Diabetes: 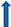 rates of ICU admission.
Huang et al. [78]	Retrospective cohort study	⋅ 299 patients with COVID-19 ⋅ Male: 53.5% ⋅ Age: 53.4 ± 16.7 years ⋅ Co-morbidities: 33.1%	⋅ Hypertension: 24.7% ⋅ Diabetes: 11.9%	⋅ Hypertension: 38.7% of the patients with hypoalbuminemia (vs. 17.1 % with normal albumin levels). ⋅ Diabetes: 22.6% and 5.7%, respectively.
Bassoli et al. [80]	Retrospective cohort study	⋅ 207 patients with COVID-19 ⋅ Male: 68.6% ⋅ Age (IQR): 60.90 (49.17–71.29) years	⋅ Hypertension: 34.3% ⋅ Diabetes: 13.5%	⋅ Hypertension: 43.8% of the patients with hypoalbuminemia (vs. 24.5% with normal albumin levels). ⋅ Diabetes: 17.1% and 9.8%, respectively.
Hundt et al. [81]	Retrospective cohort study	⋅ 1827 patients with COVID-19 ⋅ Male: 53.0% ⋅ Age: 64.6 ± 18.2 years	⋅ BMI: 29.8 ± 7.8 kg/m^2^ ⋅ Diabetes: 39.0% ⋅ Obesity: 42.5%	⋅ Hypoalbuminemia occurred in 56.7% of patients. ⋅ Hypoalbuminemia was statistically associated with BMI but not with diabetes.
Chen et al. [82]	Retrospective cohort study	⋅ 482 patients with COVID-19 ⋅ Male: 50.4% ⋅ Age (IQR): 56 (39–67) years	⋅ Hypertension: 25.3% ⋅ Diabetes: 14.9%	⋅ 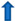 prevalence of diabetes and hypertension among patients with hypoalbuminemia.
Viana et al. [83]	Retrospective cohort study	⋅ 609 patients with COVID-19 ⋅ Male: 60.3% ⋅ Age (IQR): 71 (58–82) years	⋅ BMI (IQR): 28.90 (25.94–32.04) kg/m^2^ ⋅ Hypertension: 55.0% ⋅ Diabetes: 25.3% ⋅ Dyslipidemia: 43.5%	⋅ 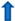 incidence of diabetes and hypertension among non-survivors when compared to survivors.
Hirashima et al. [84]	Retrospective cohort study	⋅ 61 patients with COVID-19 ⋅ Male: 65.6% ⋅ Age: 47.51 (20–88) years	⋅ BMI: 23.2 (16.9–38.9) kg/m^2^ ⋅ Hypertension: 23.0% ⋅ Diabetes: 14.8%	⋅ 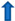 prevalence of diabetes and hypertension among critically ill patients when compared to milder cases.

ICU; intensive care unit, IMW; intermediate care ward, BMI; body mass index.

## Data Availability

Data sharing not applicable.

## Abbreviations

ACE2; Angiotensin-Converting Enzyme 2, AGEs; advanced glycation end products, Ang-2; angiopoietin-2, ARDS; acute respiratory distress syndrome, BMI; body mass index, CRP; C-reactive protein, CXCL10; CXC-chemokine ligand 10, dipeptidyl peptidase 4-inhibitors; DDP-4is, eNOS; endothelial nitric oxide synthase, ET-1; endothelin 1, FFAs; free fatty acids, glucagon-like peptide 1 receptor agonists; GLP-1RAs, HLH; hemophagocytic lymphohistiocytosis, human umbilical vein endothelial cells; HUVECs, ICAM; intercellular adhesion molecule, IL; interleukin, IMW; intermediate medicine ward, ISCLS; Idiopathic systemic capillary leak syndrome, left ventricular ejection fraction; LVEF, MAPK/ERK; mitogen-activated protein kinase/extracellular signal-regulated kinase, MCP-1; Monocyte chemoattractant protein-1, MDA; malondialdehyde, MGUS; monoclonal gammopathy of undetermined significance, NETs; neutrophil extracellular traps, NF-kB; nuclear factor kappa B, NLR; neutrophil-to-lymphocyte ratio, NLRP3; NOD-like receptor family pyrin domain-containing 3, NO; nitric oxide, OR; odds ratio, PBR; perfused boundary region, PKC; protein kinase C, PVAT; Perivascular adipose tissue, RAAS; renin-angiotensin-aldosterone system, ROS; reactive oxygen species, SFAs; saturated fatty acids, SMD; standard mean difference, SOD; superoxide dismutase, TMPRSS2; transmembrane Serine Protease 2, TNF-a; tumor necrosis factor-α, VCAM; vascular cell adhesion molecule, VEGF; vascular endothelial growth factor, WBC; white blood cell.
